# Development and validation of the information-motivation-behavioural skills model-based human immunodeficiency virus education kit for adolescents in Malaysia

**DOI:** 10.1038/s41598-024-57593-y

**Published:** 2024-03-22

**Authors:** Wan Nur Syamimi Wan Mohamad Darani, Aimi Nadira Mat Ruzlin, Zahir Izuan Azhar, Xin Wee Chen

**Affiliations:** 1 Department of Public Health Medicine, Faculty of Medicine, Universiti Teknologi MARA, Sungai Buloh, Malaysia; 2 Training Management Division, Ministry of Health, Putrajaya, Malaysia; 3 Institute of Medical Molecular Biotechnology (IMMB), Faculty of Medicine, Universiti Teknologi MARA, Sungai Buloh, Malaysia

**Keywords:** Infectious diseases, Patient education, Public health

## Abstract

The growing Human Immunodeficiency Virus (HIV) incidences and insufficient HIV knowledge among Malaysian late adolescents necessitate immediate attention to HIV prevention via education. This study aims to develop and validate an Information-Motivation-Behavioural skills (IMB) model-based education kit for adolescents, PREM-Kit, to educate on HIV prevention among Malaysian late adolescents. Utilizing the Analysis, Design, Development, Implementation, and Evaluation model, we conducted the study in three phases: needs assessment, development of PREM-Kit, and validation of PREM-Kit by applying the IMB model to map the PREM-Kit’s contents. PREM-Kit, developed in Malay language, consisted of an infographic flip chart and videos. Five multi-disciplinary experts validated the PREM-Kit using the content validity index (CVI), and 13 end-users validated the PREM-Kit using the Malay version of the Patient Education Materials Assessment Tool for Printable and Audiovisual Materials. The infographic flip chart comprised three modules covering 15 topics, and an animated video accompanied each module. PREM-Kit achieved CVI scores of 1.0 and median scores of over 80% for understandability and actionability. Overall, the newly developed IMB model-based HIV education kit has good content validity, is simple to comprehend and apply, and is ready for testing its effectiveness in improving adolescents’ knowledge, attitudes, and practices for HIV prevention.

## Introduction

Human immunodeficiency virus (HIV) is a prominent global health concern^1^. HIV, ranking among the top ten global health threats, necessitates significant focus, particularly among the younger population^2^. There is a global increase in the proportion of adolescents (aged 10–19 years) who are HIV-positive, which accounts for 10% of newly diagnosed adult HIV infections in 2022^3^. In Malaysia, more than 70% of newly reported HIV infections occurred among individuals aged 20 to 39 (working age group) in 2021, with adolescents comprising one in every 25 new people living people with HIV (PLHIV)^4^. In addition, a recent nationwide survey revealed that 86% of 15- to 24-year-olds lacked adequate knowledge regarding HIV, with 79% of those adolescents enrolled in tertiary education^5^.

Adolescents shall receive HIV education to acquire the knowledge and skills necessary to maintain safer behaviours to prevent HIV infections^6^. A more comprehensive approach to HIV education in Malaysia necessitates the inclusion of high-quality health information, such as age-appropriate comprehensive sexual education, efficient health services, and a supportive environment^7^. It has been demonstrated that an education incorporating theory-based content increases adolescents’ engagement during learning and results in favorable behavioural outcomes^6^, as it provides relatively simple explanation for complex health behaviour and identifies specific individual determinants to engage in a given health behavior^8–10^. An extensively applied behavioural theory that has demonstrated efficacy in the realm of HIV education is the Information-motivation-behavioural skills (IMB) model—which consists of three essential determinants that influence preventive behaviour: (i) information and knowledge for HIV prevention, (ii) motivation to prevent HIV infection, and (iii) behaviour skills to perform HIV prevention behaviour^11^. Similarly, the present study employed the IMB model for the content development of an HIV education kit.

In Malaysia, the primary focus of HIV education about sexual reproductive health is on school-aged children^12^. Conversely, there is often a lack of attention given to students in higher education, specifically late adolescents enrolled in pre-university programs^5,13,14^. While it is essential to provide HIV and sexual education at ealy stage of life, the delivery of age-appropriate information needs to be continuously given to late adolescents, as they require updated information necessary for their age group so that it will complement whatever existing information they received in school^15^. Besides the Healthy Youth Program Running Without AIDS (or *Program Sihat Tanpa AIDS Untuk Remaja*, PROSTAR), other existing programs i.e, PEERS—Reproductive Health and Social Education, the Young Doctor Program, and PROSIS (a program for students in higher institutions), have unstandardized contents that address only basic information on adolescents’ risk behaviours or healthy lifestyles and require many resources to maintain sustainability^16–18^. Therefore, the development of HIV educational material for late adolescents in the country is necessary, to address more sensitive issues related to HIV that are not openly discussed in the school curriculum while taking into account of socio-cultural norms and values^19^.

An optimal development process for HIV educational materials can be accomplished by applying the Analysis, Design, Development, Implementation, and Evaluation (ADDIE) instructional design model^20^. The ADDIE model, consisting of five methodical phases, has been implemented across different fields of education to design and develop curricula^20–23^. In this study, the ADDIE model was applied to guide the development of an HIV education kit, from need assessment phase to validation phase. Furthermore, it has been demonstrated that educational materials that integrate multimedia elements, including animations, images, audio, and video, contribute to educational excellence and enhance student performances ^24^.

Educational materials must be evaluated before disseminating to the intended population^25^. Content validation is a critical component in developing impactful educational materials^25^. This procedure evaluates the material's representativeness by ensuring it accurately represents the target population and eliminates unnecessary components^26^. Validating the content of educational materials can be achieved through multiple approaches, one of which involves assessing its understandability and actionability^27^. The concept of understandability pertains to the capacity of individuals with diverse health literacy levels and backgrounds to comprehend educational materials and extract essential messages. On the other hand, actionability denotes the ability of individuals to identify practical steps that can be taken in response to the information presented in the educational materials^27^. Thus, the present study aims to develop and validate a new IMB model-based HIV education kit, in the Malay language (the Malaysia national language), namely, PREM-Kit (*Kit Pencegahan HIV di Kalangan Remaja*—a Malay language acronym) for adolescents in Malaysia.

## Methods

**Figure 1:**
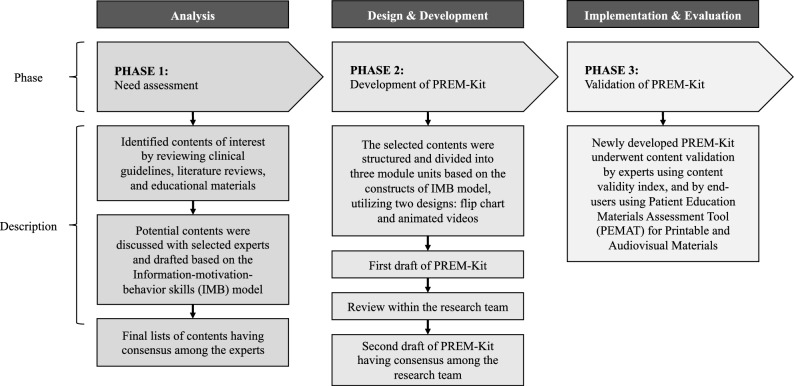
Overview of study phases. The figure shows the development and validation phases of PREM-Kit based on ADDIE model (white font).

The present study comprised three phases, namely, (i) need assessment, (ii) development of PREM-Kit, and (iii) validation of PREM-Kit, which were adapted following the ADDIE model (Fig. 1)^21,22^. The study was conducted between July 2022 and February 2023. It was reviewed and approved by the Universiti Teknologi MARA (UiTM) Research Ethics Committee (REC/05/2022 (ST/MR/85)) and Medical Research Ethics Committee, Ministry of Health, Malaysia (RSCH ID-22-01243-BV2).

### Phase 1—need assessment

The first phase focused on identifying the contents of interest for the PREM-Kit. We studied all related clinical guidelines, literature reviews, and existing educational materials (i.e, poster, pamphlet, booklet, video, and online module) relevant to HIV and adolescents from recognized institutions and organisations ^15,17–19,28,29^. Potential contents were extracted and summarized in a PowerPoint slide, which was subsequently discussed using the working group technique through a series of online meetings (via the Google Meet platform) with a group of experts with more than 5 years of experience who are specialized in HIV management or infectious diseases or health behaviour and education field^25,30^. The contents were originally drafted in the Malay language based on the IMB model to form the final list of topics. Overall, two rounds of expert’ discussion were conducted, with each expert providing individual inputs using a feedback form emailed separately following each session^30^.

### Phase 2—development of PREM-Kit

In the development phase, we designed two types of HIV educational materials with multimedia elements to deliver PREM-Kit’s contents: (i) printed material in the form of an infographic flip chart and (ii) audiovisual materials in animated videos. Both materials were designed and comprised three modules that were organized according to the construct of IMB model—the first module describes HIV facts and information, the second module explains the motivation for HIV prevention, and the third module demonstrates behavioural skills for HIV prevention. The selected topics for the flip chart were initially drafted in a PowerPoint slide and were subsequently converted into infographic forms using the Canva graphic design platform (Canva Pty Ltd—canva.com). We collaborated with experts from the College of Creative Arts, UiTM, to develop three animated videos—one for each module—using Powtoon, an online amination software (Powtoon Ltd—powtoon.com) based on the principles and guidelines for maximizing student learning from video content^31^. Several rounds of review and revision of both materials were carried out among the research team until a consensus was reached.

### Phase 3—validation of PREM-Kit

The present study used two validation processes: (i) expert validation and (ii) end-user validation to evaluate the PREM-Kit. Both validation processes were performed online, and all communications were conducted through emails, phone calls, and text messages (via WhatsApp). All participants were provided with a link to access a Google Drive folder comprising (i) evaluation forms (according to types of validation) and (ii) two other folders containing flip charts in PDF format and videos labelled according to modules.

### Expert’s validation

Five healthcare professionals (experienced in HIV management) were recruited to independently review and validate both materials using the content validity index (CVI) based on relevant aspects, with a 4-point Likert scale rating: score 1 = not relevant, 2 = somewhat relevant (submodule needs revision), 3 = relevant but needs minor revision, 4 = very relevant^25^. Following these ratings, the scale was further categorized into relevant content (ratings 3 and 4) and nonrelevant content (ratings 1 and 2)^25,32^. To obtain the final score of CVI, we measured: (i) item-CVI (I-CVI), which was computed as the number of experts who rate “relevant content” for each topic/video, divided by the total number of experts, and (ii) scale-CVI (S-CVI/Ave), an average of the I-CVI for all topics/videos. The content was considered appropriate if the CVI was greater than 0.79; it required revision if the CVI was between 0.70 and 0.79; and it was eliminated if the CVI was less than 0.70^25,33^.

### End-user’s validation

The contents of PREM-Kit were further evaluated pertaining to its understandability and actionability of each material (flip chart and videos)^27^. In this process, we recruited several end-users (i.e. a person who will use the PREM-Kit when it is published) based on the following criteria: (i) health care workers involved in HIV management or counsellor or lecturer or adolescent aged 18–19 years, and (ii) a person who is fluent in Malay language (speaking and writing), to validate the content of PREM-Kit. All participants were given two evaluation forms to assess both materials. The Malay language version of Patient Education Materials Assessment Tool (PEMAT) for Printable Materials (PEMAT-P) was used to assess the understandability and actionability of the flip chart through its 17 items for understandability and seven items for actionability. Concurrently, the Malay version of PEMAT for Audiovisual Materials (PEMAT-A/V) consisting 13 items for understandability and four items for actionability was used to evaluate the videos. PEMAT-P and PEMAT A/V use scoring as either 1 (agree), 0 (disagree), or N/A (not applicable to the material). The total scores for understandability and actionability were calculated and displayed as a percentage (%). Higher understandability and actionability scores suggest that the education material is simple to comprehend and implement^34^.

### Ethics statement

This study was reviewed and approved by the Universiti Teknologi MARA Research Ethics Committee (REC/05/2022 (ST/MR/85)) and the Medical Research Ethics Committee, Ministry of Health, Malaysia (RSCH ID-22-01243-BV2). We conducted all methods in this study in accordance with the guidelines outlined in the Declaration of Helsinki. Informed consent was obtained from all participants prior to the commencement of the study.

## Results

### Phase 1—need assessment

**Figure 2:**
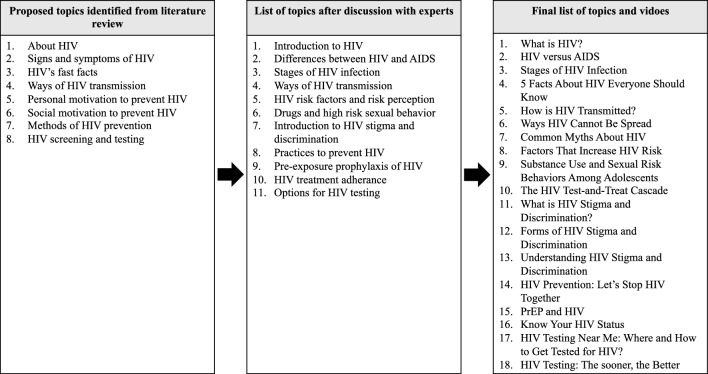
Need assessment of PREM-Kit’s contents. The figure illustrates how PREM-Kit’s contents are generated during need assessment.

Six experts recruited in this phase were from different disciplines working with PLHIV, they were infectious disease specialist, HIV microbiologist, family medicine specialist, adolescent psychologist, social behaviourist, and health education officer. Majority of them were female (67%) with median (interquartile range) duration of service of 14 (2.8) years (Table 1). Figure 2 shows the process for generating topics for PREM-Kit via a series of online discussions with the experts. Based on the literature search, eight main topics were identified, proposed, and discussed in the first session with the experts, subsequently after a series of online discussions, the proposed topics were expanded, with 11 generated, followed by a final list of a total of 18 topics, including three videos approved to be included in the PREM-Kit. All experts agreed that the contents of the PREM-kit should focus on three main modules, namely: (i) HIV knowledge, (ii) personal and social motivation to prevent HIV, and (iii) empowering adolescents with HIV preventive measures and skills. We also included suggestions to incorporate information on drug use and its relationship with high-risk sexual behaviours in adolescents, options for HIV testing, including self-testing, introduction to preexposure prophylaxis (PrEP), and HIV treatment cascade.

**Table 1:** Socio-demographic characteristics of study participants.

Characteristics	Need assessment (N = 6)		Expert’s validation (N = 5)		End-user’s validation (N = 13)	
	n (%)	Median (IQR)	n (%)	Median (IQR)	n (%)	Median (IQR)
Age^a^						36 (11.0)
Gender						
Male	2 (33.0)		1 (20.0)		3 (23.1)	
Female	4 (67.0)		4 (80.0)		10 (76.9)	
Ethnicity						
Malay	4 (67.0)		4 (80.0)		11 (84.6)	
Chinese	2 (33.0)		1 (20.0)			
Indian					1 (7.7)	
Bumiputera Sabah					1 (7.7)	
Profession						
Infectious disease specialist	1 (16.7)		1 (20.0)			
HIV microbiologist	1 (16.7)		1 (20.0)			
Family medicine specialist	1 (16.7)		1 (20.0)			
Adolescent psychologist	1 (16.7)		1 (20.0)			
Social behaviorist	1 (16.7)					
Health education officer	1 (16.7)		1 (20.0)			
Lecturer					2 (15.4)	
Counselor					2 (15.4)	
Paramedic					4 (30.8)	
Assistant environmental health officer					2 (15.4)	
Late adolescent					3 (23.0)	
Duration of services (years)		14 (2.8)		15 (2.0)		

*HIV* human immunodeficiency virus, *IQR* interquartile range.

^a^Minimum age of end-user = 18 years old, maximum age of end-user = 48 years old.

### Phase 2—development of PREM-Kit

Table 2 displays an overview of PREM-Kit, listed 15 topics developed under three modules, along with three short videos that were accompanied by either a narrator voice or an added subtitle presented in the speech bubble. All materials were in Malay language, highlighted age-appropriate contents in simple terms and language, attractive and colorful illustrations, and graphic aids in conveying complex concepts.

**Table 2:** Overview of PREM-Kit.

Module	Descriptions	Contents		Method of delivery	No. of Pages/duration of video (min)
Module 1: HIV facts and information	Module 1 is based on the information construct (I) of the IMB model. It describes HIV facts and basic information and the differences between HIV facts and myths. There are six topics included in the flip chart and an animated video on HIV facts versus HIV myths	T1	What is HIV?	Flip chart	6
		T2	HIV versus AIDS		
		T3	Stages of HIV infection		
		T4	5 Facts about HIV everyone should know		
		T5	How is HIV transmitted?		
		T6	Ways HIV cannot be spread		
		V1	Common myths about HIV	Video demonstration	02:51
Module 2: motivation for HIV prevention	Module 2 is based on the motivation construct (M) of the IMB model. This module describes, (i) personal motivation for HIV prevention related to HIV risk perception and (ii) social motivation – basic information on HIV stigma and discrimination. It includes five topics in the flip chart and a video highlighting HIV stigma and discrimination.	T7	Factors that increase HIV risk	Flip chart	5
		T8	Substance use and sexual risk behaviors among adolescents		
		T9	The HIV test-and-treat cascade		
		T10	What is HIV stigma and discrimination?		
		T11	Forms of HIV stigma and discrimination		
		V2	Understanding HIV stigma and discrimination	Video demonstration	01:28
Module 3: behavior skills for HIV prevention	Module 3 is based on the third construct of the IMB model which is behavioral skills (B). This module describes methods for HIV prevention and provide brief explanations of HIV screening test, who should get tested? and the procedure of HIV screening. It includes four more topics in the flip chart and a video to promote HIV testing among adolescents.	T12	HIV prevention: Let’s stop HIV together	Flip chart	4
		T13	PrEP and HIV		
		T14	Know your HIV status		
		T15	HIV testing near me: Where and how to get tested for HIV?		
		V3	HIV testing: The sooner, the Better	Video demonstration	01:25

*HIV* human immunodeficiency virus, *IMB* information-motivation-behavioral skills model, *PrEP* pre-exposure prophylaxis, *T* topic, *V* video.

### Phase 3—validation of PREM-Kit

A total of five experts and 13 end-users validated the PREM-Kit. Table 3 showed the agreement ratings of experts, along with the I-CVI and S-CVI scores for the contents of PREM-Kit. Overall, all contents were relevant, based on the excellent scores of both I-CVI and S-CVI (1.0). Minor comments such as general formatting, paragraphing, and sentence structure were received, thereafter revisions were made accordingly.

**Table 3:** Scores of PREM-Kit’s relevant rating by experts.

Module	Topic/video	Experts in agreement (n = 5)		CVI^a^	Interpretation
		Relevant (rating 3 or 4)	Non-relevant (rating 1 or 2)		
Module 1: HIV facts and information	T1	5		1	All topics and video are relevant
	T2	5		1	
	T3	5		1	
	T4	5		1	
	T5	5		1	
	T6	5		1	
	V1	5		1	
Average CVI (S-CVI/Ave^b^)				1	
Module 2: motivation for HIV prevention	T7	5		1	All topics and video are relevant
	T8	5		1	
	T9	5		1	
	T10	5		1	
	T11	5		1	
	V2	5		1	
Average CVI (S-CVI/Ave^b^)				1	
Module 3: behavior skills for HIV prevention	T12	5		1	All topics and video are relevant
	T13	5		1	
	T14	5		1	
	T15	5		1	
	V3	5		1	
Average CVI (S-CVI/Ave^b^)				1	

*CVI* item content validity index, *HIV* human immunodeficiency virus, *IMB* information-motivatio-behavioral skills model, *S-CVI/Ave* scale content validity index, *T* topic; *V* video.

^a^I-CVI: item-CVI. It is calculated by the number of experts who rated the topic/video as relevant (rating 3 or 4, divided by the total number of experts.

^b^S-CVI/Ave: scale-CVI. It is calculated by the sum of I-CVI score, divided by the total number of topic/video.

Table 4 presents the end-user’s validation findings by assessing the understandability and actionability of the PREM-Kit. The median scores reported for the flip chart and videos were greater than 80%. Both materials were found to be visually appealing, straightforward, and simple to comprehend.

**Table 4:** Scores of the understandability and actionability of PREM-Kit by end-users.

PREM-Kit’s component	Understandability score (%)			Actionability score (%)		
	Median (IQR)	Min^a^	Max^b^	Median (IQR)	Min^a^	Max^b^
Flip chart	100.0 (5.9)	93.8	100.0	100.0 (0.0)	83.3	100.0
Video 1	100.0 (0.0)	90.0	100.0	100.0 (0.0)	–	–
Video 2	100.0 (0.0)	90.0	100.0	100.0 (0.0)	–	–
Video 3	100.0 (0.0)	92.3	100.0	100.0 (0.0)	–	–

*IQR* interquartile range, *Max* maximum score, *Min* minimum score.

^a^Min: represent the lowest score obtained from all the end-users (n = 13). It is calculated by dividing total scores with maximum marks (in percentage).

^b^Max: represent the highest score obtained from all the end-users (n = 13). It is calculated by dividing total scores with maximum marks (in percentage).

## Discussion

HIV continues to be a significant global health concern, with adolescents being a vulnerable population, including in Malaysia^35^. In the present study, we successfully developed an HIV education kit (PREM-Kit) which was (i) grounded with a theoretical framework of IMB model, (ii) has good content validity, and (iii) easily understood and implemented contents delivered through containing an infographic flip chart and animated videos.

PREM-Kit demonstrates optimal validation scores which implies the excellent relevance of the contents. The comprehensive and iterative processes in developing PREM-Kit to identify knowledge gaps, misconceptions, and timely HIV preventive measures, as well as collaboration with experts in HIV, educators, and adolescents, contribute to the formation of its accurate and engaging contents. This result aligns with the conclusions of other studies using similar approaches, which have demonstrated that educational materials across various health fields possess satisfactory validation scores^36,37^. A satisfactory agreement between experts implied that the developed module effectively accomplishes its goals to strengthen HIV knowledge among the late adolescents in Malaysia and their motivation to prevent HIV, therefore empowering this population in practicing good HIV preventive behaviours. This result could be attributed to applying the IMB model when framing the contents of PREM-Kit, which is consistent with other studies employing a similar theoretical framework^38–41^.

One of the strengths of our PREM-Kit is it uses multimedia elements such as infographic flip chart and animated videos to deliver its content to the target population. It has been demonstrated that the use of these elements aids in information processing, which helps adolescents comprehend abstract concepts or memorize information better^24,42,43^. Furthermore, during validation, our PREM-Kit showed high scores of understandability and actionability domains, based on the ratings given by end-users, indicating that it is easily comprehensible and effectively communicates with its audience and outlines concrete actions they could adopt, which is consistent with previous research using similar delivery method^37,44,45^. In contrast to prevailing peer-based programs that necessitated trainer and facilitator training for implementation and upkeep, we assert that our PREM-Kit operates independently and is user-friendly for a diverse array of end-users in various contexts. Hence, it would serve as a valuable supplement to these established programs in the country. On top of that, our videos are animated, which is the preferred characteristic of educational videos for Malaysian adolescents compared to realism and minimalist themes^45^. Each of the PREM-Kit’s videos was short in duration, lasted for less than three-minute-long and were meticulously produced in accordance with suggestions by the College of Creative Arts experts. These designs stimulate the cognitive development of the intended audience (adolescents) by grabbing their interest and decreasing mind wandering to maximize their learning from the video content^31^.

Besides, PREM-Kit also delivers HIV education to adolescents in our native language (Malay), which is also the official language of the country. Approximately, 95.3% of Malaysian adolescents prefer for health education materials in their native language^45^; this preference is believed to result from the use of Malay as an intermediate language in educational institutions, which serves as a common language for all students^46^. It is challenging to accomplish the learning objectives when students attempt to acquire or are compelled to adopt a language that is not their first^47^. This is especially true when they are gaining new knowledge through education. They could find it hard to explain themselves and make sense of the information^47^. Adolescents will, therefore, benefit from HIV education delivered in their native language since it will eliminate language barriers and promote inclusivity and high-quality learning, all of which will enhance educational outcomes^45,48^.

Besides this, several challenges were encountered throughout three phases, however, successfully solved. Working group technique (in the first phase) was selected because it is flexible, relatively easy to set up and can generate insights and shared ideas from a selected group of individuals about their views and experiences on a topic, as compared to other techniques, such as interviews and surveys, which may have influenced responses from the interviewer resulting in bias and having limited amount of information beyond those inquiries articulated in a survey^30,36,44,45^. However, managing a group discussion remains a challenge as we need to coordinate practical time and logistic arrangements that meet the needs of our experts. Moreover, we need to maintain equal participation from each expert^30^. To overcome this, we included a separate individual feedback response after each group discussion to reduce the influence of group opinion on the individual expert^30,49^.

Despite achieving optimal CVI scores and receiving excellent scores in the understandability and actionability domains, the present study is lacking in qualitative input from the adolescents which would provide different perspectives from adolescents that resonate with the target population. Nevertheless, the need assessment for this study was carried out utilising data sources and references recommended by credible institutions and organisations, complemented with inputs based on discussions with professionals who are experts in the respective field^29^. We opine that a regular update and revision of the content of PREM-Kit from time to time. For instance, the Malaysia HIV self-testing guideline recently launched in August 2023 has highlighted the principles of 5Cs when introducing self-testing—consent, confidentiality, conselling, correct results and connected^50^. Therefore, future studies could explore on more recent issues, beliefs, and behaviours of adolescents related to HIV. Also, more surveys on the awareness and acceptance of PrEP among adolescents shall be conducted in Malaysia. This is important because it is evident that PrEP plays a crucial role in HIV prevention among Malaysian adolescents^35,51^, however, little is known on the acceptance given that majority Malaysians is Muslim and various facets of society and culture perceptions^52–54^.

In future, we recommend further studies to test the effectiveness of this PREM-Kit in improving the knowledge, attitudes, and practices for HIV prevention among the adolescents. Such evaluation is important to inform further decisions and future plans for the kit’s distribution, which may include integration into students’ curriculum in higher education, such as HIV education via the PROSIS program, and also incorporating the use of this kit in adolescents’ health clinics and non-governmental adolescents’ centres to catch drop-out school adolescents. Additionally, a continuous update of information is essential to ensure the sustainability of the kit and relevancy to latest HIV guideline and policy in Malaysia.

## Conclusion

A comprehensive and iterative process adapted from the ADDIE model in developing an IMB model-based HIV education kit led to the formation of contents with strong relevance. Validation from multi-disciplinary experts and end-users greatly supported the delivery of PREM-Kit to adolescents in giving valuable insights into the kit’s ultimate benefits. This newly developed HIV education kit is validated, ready for testing its effectiveness in improving the knowledge, attitudes, and practices for HIV prevention among the adolescents, yet regular update of PREM-Kit is essential.

## Data Availability

All data generated or analyzed during this study are included in this published article.
